# Dietary Taxifolin Protects Against Dextran Sulfate Sodium-Induced Colitis *via* NF-κB Signaling, Enhancing Intestinal Barrier and Modulating Gut Microbiota

**DOI:** 10.3389/fimmu.2020.631809

**Published:** 2021-02-16

**Authors:** Jinxiu Hou, Mingyang Hu, Le Zhang, Ya Gao, Libao Ma, Qingbiao Xu

**Affiliations:** College of Animal Sciences and Technology, Huazhong Agricultural University, Wuhan, China

**Keywords:** colitis, gut microbiota, intestinal barrier, inflammatory bowel disease, NF-*κ*B, taxifolin

## Abstract

Taxifolin is a natural antioxidant polyphenol with various bioactivities and has many beneficial effects on human gut health. However, little is known of its function on colitis. In this study, the protective effects of taxifolin on colitis symptoms, inflammation, signaling pathways, and colon microbiota were investigated using dextran sulfate sodium (DSS)-induced colitis mice. Intriguingly, pre-administration of taxifolin alleviated the colitis symptoms and histological changes of the DSS-challenged mice. Supplementation of taxifolin significantly inhibited the secretions of tumor necrosis factor-α, interleukin (IL)-1β, and IL-6 and significantly increased the secretions of IL-10, secretory immunoglobulin A, superoxide dismutase, and immunoglobulins (IgA, IgG, and IgM) in DSS-induced colitis mice. In addition, the activation of nuclear factor kappa B (NF-*κ*B; p65 and I*κ*B*α*) signaling was significantly suppressed by taxifolin supplementation. The expression of tight junction proteins (claudin-1 and occludin) was significantly increased by taxifolin. Moreover, 16S rDNA sequencing revealed that the DSS-induced changes of colon microbiota composition and microbial functions (amino acid metabolism and MAPK signaling) were restored by taxifolin, including the decreases of the abundances of *Bacteroides*, *Clostridium ramosum*, *Clostridium saccharogumia*, *Sphingobacterium multivorum*, and the ratio of *Bacteroidetes*/*Firmicutes*, and the increases of the abundances of *Desulfovibrio C21 c20* and *Gemmiger formicilis* at species level. In conclusion, these results revealed that dietary taxifolin has a great potential to prevent colitis by inhibiting the NF-*κ*B signaling pathway, enhancing intestinal barrier, and modulating gut microbiota.

## Introduction

Inflammatory bowel disease (IBD) is caused by inflammation and oxidative stress in the colon with the symptoms of gut bleeding, diarrhea, and body weight (BW) loss (1). The antigen invasion can activate the mucosal immune response, resulting in the release of proinflammatory cytokines from macrophages, such as tumor necrosis factor (TNF)-α, interleukin (IL)-1β, IL-6, and IL-8, which can exacerbate inflammatory severity and colitis (1). However, the use of drugs to treat IBDs is often associated with undesirable side effects (2). Recently, the replacement of drugs by dietary polyphenols derived from plants comes into people’s view (3).

As a common bioactive component, polyphenol can benefit gut health *via* modulating barrier functions, signaling pathways, immune responses, and gut microbiota compositions (4). Various studies have been carried out to investigate the protective and therapeutic effects of dietary polyphenols on dextran sodium sulfate (DSS)-induced mice colitis (3), such as myricitrin (2), procyanidin (5), phytosteryl ferulates (6), ellagic acid (7), peracetylated (−)-epigallocatechin-3-gallate (AcEGCG) (8), naringenin (9), chlorogenic acid (10), anthocyanin, rosmarinic acid (11), magnolol (12), and resveratrol (13). Bioactive polyphenol taxifolin (3,5,7,3,4-pentahydroxy flavanone or dihydroquercetin) is a Chinese traditional medicinal herb and can be extracted from onions, grapes, olive oil, citrus fruits, sorghum grain, milk thistle, and artichoke (14, 15). It has been reported that taxifolin displays various bioactivities, including antioxidative, anti-inflammatory, anti-microbial, anti-diabetic, antitumor, anti-allergic, hepatoprotective, and cardioprotective activities (15–17).

Taxifolin can inhibit oxidative stress and alleviate inflammation *via* inhibiting the expression and production of TNF-α and IL-6 in LPS-induced inflammatory mice (18), and can inhibit oxidative enzymes, reduce the overproduction of reactive oxygen species (ROS), and modulate nuclear factor kappa B (NF-*κ*B) activation to ameliorate injury (19, 20). Taxifolin can inhibit the expression of cyclo-oxygenase-2 (COX-2) associated with oxidative properties in rats with cerebral ischemic reperfusion injury (19). Moreover, taxifolin also can attenuate myocardial apoptosis (21) and has the protective effect on mouse liver injury *via* decreasing the expression of TNF-α and IL-6, and increase SOD expression (22). Recently, it was reported that taxifolin can alleviate iron overload-induced inflammation (23). Taxifolin has anti-inflammatory and anti-microbial activities with the therapeutic promise in cancer, cardiovascular, and liver disease (24). In addition, taxifolin can also change the abundance of gut microbiota and decrease *E. coli* abundance (25). As a promising therapeutic agent, taxifolin has attracted an increasing interest of researchers. However, the effects and mechanisms of taxifolin on colitis are still unclear. Thus, the objective of this study is to explore the potential effects of taxifolin on DSS-induced acute colitis mice.

## Materials and Methods

### Animal Groups and Administrations

Thirty male Institute of Cancer Research (ICR) mice (aged 7–8 weeks, approximately 30 ± 2 g BW) were obtained from Liaoning Changsheng Biotechnology Co., Ltd. (Benxi, China). Taxifolin was purchased from Yuanye Bio-Technology Co., Ltd. (S31601, Shanghai, China). DSS was obtained from Sangon Biotech Co., Ltd. (A600160-0250, Shanghai, China). The mice were randomly separated into three groups: Control, DSS, and taxifolin + DSS (taxifolin) group (*n* = 10). The mice from the taxifolin group received taxifolin dissolved in distilled water *via* intragastric gavage daily from day 1 to day 14 (100 mg/kg BW). On day 8, the mice from DSS and taxifolin groups were given distilled water containing 3% DSS (w/v) for additional 7 days. The dose of taxifolin is a safe dose to use in clinical studies (14). All the mice received a basal diet and were kept in cages in a room with a 12-h light and 12-h dark with controlled temperature (25 ± 2°C) and humidity (50 ± 5%). On day 15, all the mice were sacrificed. The animal experiment was performed under the guidelines of the Laboratory Animal Ethics Committee of Huazhong Agricultural University.

### Disease Activity Index

The DAI of the mice was evaluated daily after the DSS treatment as the sum of the scores from BW loss (0–3, 0 to 10% loss), stool consistency (0–3, normal to diarrhea), stool bleeding (0–3, no to all blood in stool), and mice condition (0–3, normal to poor condition) (26).

### Histologic Analysis of Mice Colon

After the mice were sacrificed, the colon length was measured, and the distal colon (~3.5 cm proximal to anus) was washed using ice cold phosphate-buffered saline (PBS). Subsequently, the tissue samples were fixed with 4% paraformaldehyde, dehydrated in ethanol, and embedded in paraffin. A 5-μm section was prepared and stained with hematoxylin and eosin (H&E). Finally, colon morphology was obtained using a light microscopy. The extent of tissue damage was scored according to the degrees of inflammatory cell infiltration (0–3, rare to transmural extension) and tissue damage (0–3, no to extension) as described in a previous report (26).

The apoptotic level of colon cells was assessed using terminal deoxynucleotidyl transferase dUTP nick end labeling (TUNEL) assay by an *in situ* cell death detection kit (Roche, Basel, Switzerland) following the manufacturer’s instructions. The nuclei were stained using DAPI mounting solution and the images were observed using a fluorescent microscopy (DMIL LED, Leica, Germany).

### Enzyme-Linked Immunosorbent Assay

Mice colon tissues were homogenized in ice-cold PBS and centrifuged at 13,000*g* at 4°C for 20 min. The supernatant was collected to analyze the concentrations of secretory immunoglobulin (SIg) A, pro-inflammatory cytokine IL-10, and antioxidant index superoxide dismutase (SOD) using an ELISA kit (Nanjing Jiancheng Bioengineering Institute, Nanjing, China) based on the manufacturer’s instructions. Protein concentrations were determined using a Bicinchoninic acid Protein Assay Kit (BCA, KeyGEN Biotech, Nanjing, China). The results were normalized to the protein concentration of each sample. In addition, mice serum was separated from blood by centrifugation at 1,500 g for 15 min and then stored at −20°C before testing. The concentrations of TNF-α, IL-1β, IL-6, immunoglobulin (Ig) A, IgG, and IgM in serum were analyzed using an ELISA kit (Nanjing Jiancheng Bioengineering Institute, Nanjing, China) and measured using a Microplate Reader (Bio-Rad, 680, Hercules, CA, USA) at 450 nm. Each sample was tested in duplicate.

### Western Blot

Colon tissues were homogenized with a Radio Immunoprecipitation Assay buffer solution for 30 min at 4°C and centrifuged at 12,000 r for 10 min. The content of the supernatant protein was determined using a BCA kit. Then, the protein sample was added to the corresponding proportion of SDS gel loading buffer and boiled to denature for 10 min. The protein samples were separated by 10% sodium dodecyl sulfate-polyacrylamide gel electrophoresis (SDS-PAGE) at 80 V for 1 h and at 120 V for another 2 h and then were transferred to PVDF membranes (Sigma-Aldrich, UK), which were then blocked with 5% BSA (Sangon Biotech, Shanghai, China) for 2 h. After washing with TBST solution, the membranes were incubated with primary antibodies at 4°C overnight, including antibodies against *β*-actin (CST 4970S, Cell Signaling Technology, Danvers, MA, USA), NF-κB p65 (CST 6956T), pp65 (AP0124, Santa Cruz, CA, USA), I*κ*B*α* (CST 4814S), pI*κ*B*α* (CST 2859S), extracellular signal-regulated kinase (ERK; CST 4695S), pERK (CST 4370S), p38 (CST 8690S), pp38 (CST 4511S), c-Jun N-terminal kinase (JNK; CST 3708S), pJNK (CST 9255S), claudin-1 (CST 13995S), and occludin (sc-133255, Santa Cruz, CA, USA). After washing with TBST solution, the membranes were incubated with an HRP-conjugated secondary antibody (1:8,000, CST, Danvers, MA, USA) at 37°C for 1 h. Finally, the target protein in the membranes was visualized with an enhanced chemiluminescence detection system (Biotanon, China) and Chemilmaging software (Baygene biotech, China). The intensity of the bands was analyzed using the ImageJ software (NIH, USA) and normalized to the actin protein intensity.

### Gut Microbiota Analysis

The luminal content in mice colon was collected, and the microbiota DNA was extracted by a QIAamp DNA stool Mini Kit (Qiagen, Hilden, Germany). Then, the v3–v4 regions of 16S rDNA sequence were amplified using PCR, and the products were purified and sequenced using the HiSeq platform with the original data being filtered. Pairs of reads obtained from double-end sequencing were assembled into a sequence by using overlapping relationship to obtain tags of the high-variation region. The tags with above 97% similarity were clustered into an operational taxonomic unit (OTU). The OTU representative sequence was analyzed by using the RDP classifier Bayesian algorithm, and the community composition was analyzed at the levels of phylum, class, order, genus, and species. Alpha and beta diversity analyses were carried out by Quantitative Insights into Microbial Ecology (QIIME; http://qiime.org/). Cladogram plot was drawn using Figure Tree software (http://tree.bio.ed.ac.uk/software/figtree/) to identify corresponding group biomarkers. Linear discriminant analysis (LDA) effect size (LEfSe) analyses of 16S sequences were carried out using the LEfSe tool. An LDA score >4 was used and considered to be an important contributor to the model. The function of the microflora was also predicted using the Phylogenetic Investigation of Communities by Reconstruction of Unobserved States (PICRUSt) software (http://picrust.github.com). The pre-calculated genome content was searched for each OTU for metagenome prediction to identify KEGG levels 1, 2, and 3.

### Statistical Analyses

Statistical analyses were carried out using *t*-test between two groups and one-way ANOVA followed by Tukey’s multiple comparisons among multiple groups using SPSS software (SPSS Inc., Chicago, IL, USA). The data were shown as means ± standard error of mean (SEM). A difference of *P <*0.05 was considered as statistical significance.

## Results

### Taxifolin Alleviated DSS-Induced Mice Acute Colitis

**Figure 1:**
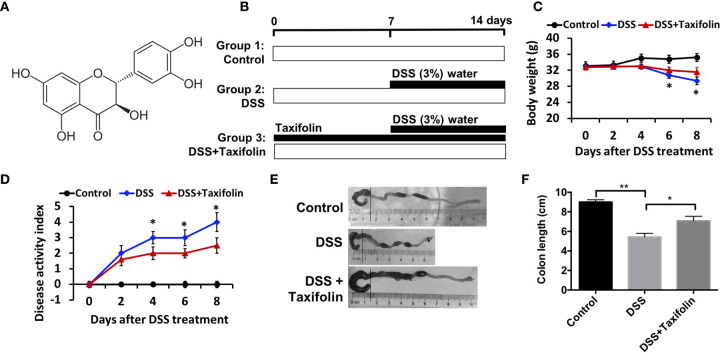
Oral administration of taxifolin alleviated DSS-induced acute colitis symptoms of mice. **(A)** Chemical structure of taxifolin; **(B)** A timeline of the experiment treatment; **(C)** Body weight loss; **(D)** Disease activity index score; **(E**, **F)** Colon length of the mice. Data were expressed as means ± SEM (*n* = 10). *P < 0.05; **P < 0.01.

Taxifolin is a polyphenol with the chemical structure as shown in **Figure 1A**. The experiment was conducted in a timeline as illustrated by **Figure 1B**. Severe acute colitis in mice has been exhibited by oral administration with 3% DSS for a week, including BW loss (**Figure 1C**), appearance of diarrhea, presence of blood in stool, and disease activity index (DAI) (**Figure 1D**) observed on day 4 after the DSS challenge. The colon length of the mice was significantly shortened by the DSS challenge (**Figures 1E, F** **)**. BW was significantly decreased after the DSS treatment and this loss was attenuated by oral administration with taxifolin (**Figure 1C**). The high DAI induced by DSS was also significantly decreased by taxifolin on day 4 after the DSS treatment (**Figure 1D**). The shortened colon length induced by DSS was significantly increased by taxifolin administration (**Figures 1E, F** **)**. In summary, these effects and colitis symptom induced by DSS challenge were dramatically attenuated by oral taxifolin supplementation (**Figure 1**).

### Taxifolin Reduced Colon Damage in DSS-Induced Mice Colitis

**Figure 2:**
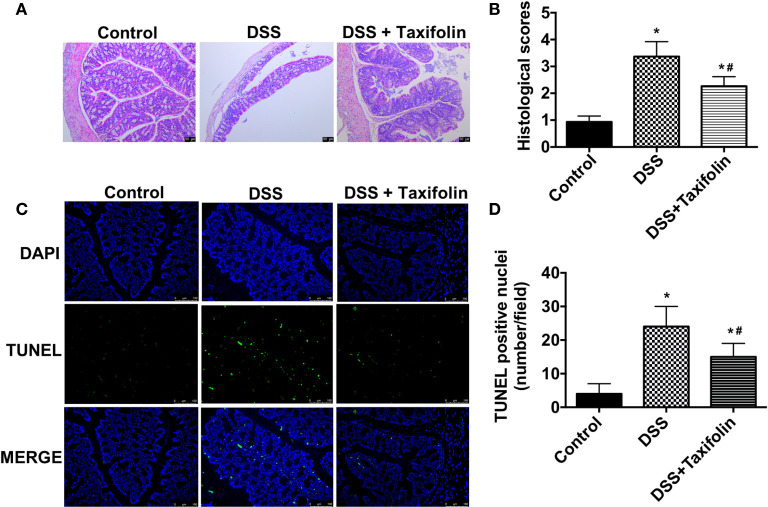
Oral administration of taxifolin alleviated DSS-induced colon histological changes of mice. **(A)** The H&E staining of the colon morphology of mice. The bars represent 50 μm. **(B)** Histological scoring was obtained from the H&E results of colon; **(C)** The fluorescent micrographs of TUNEL (green) and DAPI (blue) staining of colon. The bars represent 100 μm. **(D)** Quantitation of TUNEL positive nuclei per field obtained from fluorescent micrographs. Data were expressed as means ± SEM (*n* = 10). ^*^ *P* < 0.05 *vs*. control group, ^#^ *P* < 0.05 *vs*. DSS group.

Significant tissue damage in DSS-treated mice was shown in H&E straining images of colon tissues, including crypt loss and leukocyte infiltration. However, these damage signs were noticeably ameliorated by the supplementation of taxifolin (**Figure 2A**). The mice treated with DSS had a significantly higher colon histological index, which was significantly decreased by taxifolin supplementation (**Figure 2B**). Moreover, TUNEL positive nuclei showed that the apoptosis in colon was significantly increased by DSS challenge. However, this alteration was significantly attenuated by taxifolin supplementation (**Figures 2C, D** **)**. These results suggested that taxifolin protected colon tissue integrity and attenuated DSS-induced tissue damage.

### Effects of Taxifolin on Secretions of Cytokines, Immunoglobulins and Oxidative Indexes

**Figure 3:**
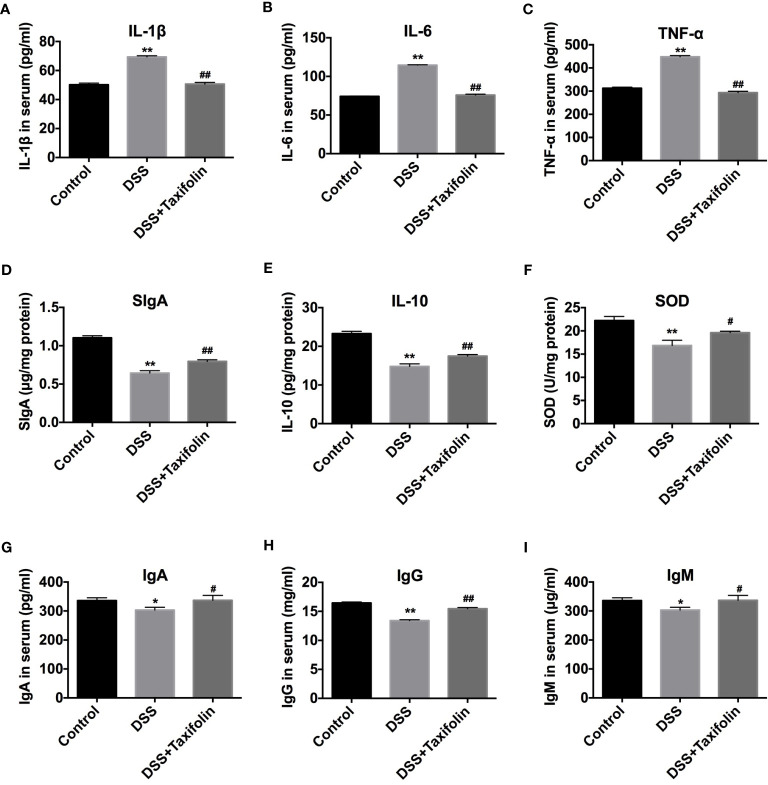
Taxifolin decreased the content of pro-inflammatory cytokines and increased anti-inflammatory cytokines and immunoglobulins of DSS-challenged mice. **(A–C)** The concentrations of IL-1β, IL-6, and TNF-α in serum; **(D–F)** Concentrations of SIgA, IL-10, and SOD in colon tissue; **(G–I)** Concentrations of IgA, IgG, and IgM in serum. Data were expressed as means ± SEM (*n* = 10). ^*^ *P* < 0.05 and ^**^ *P* < 0.01 *vs*. control group; ^#^ *P* < 0.05 and ^##^ *P* < 0.01 *vs*. DSS group.

The treatment of DSS significantly increased the concentrations of pro-inflammatory cytokines TNF-α, IL-1β, and IL-6 in mice serum (**Figures 3A–C**). DSS treatment also significantly decreased the concentrations of SIgA, anti-inflammatory cytokine IL-10, and antioxidative indicator SOD activity in colon tissues (**Figures 3D–F**) and the concentrations of immunoglobulins IgA, IgG, and IgM (**Figures 3G–I**) in the serum of the mice. However, taxifolin supplementation significantly rescued these DSS-induced changes and protected against mice colitis (**Figure 3**).

### Western Blotting Analysis

**Figure 4:**
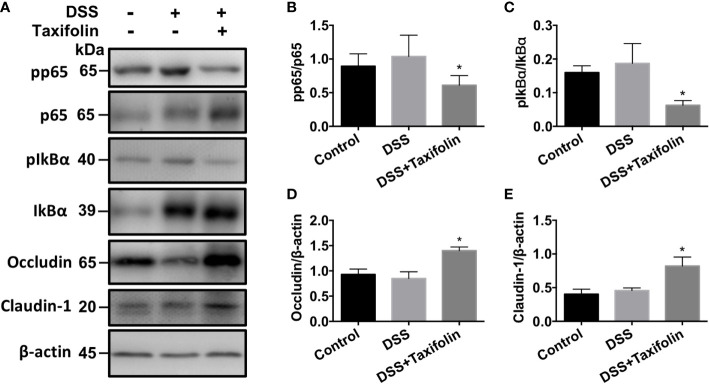
Taxifolin inhibited the NF-κB signaling and increased tight junction protein expressions in colon tissues of mice. **(A)** Representative protein bands for pp65, p65, pIκBα, IκBα, occludin, and claudin-1; **(B–E)** Statistical analysis of protein bands. Data were expressed as means ± SEM (*n* = 5). ^*^ *P* < 0.05 *vs*. DSS group.

The protein expressions of phosphorylation NF-*κ*B p65 (**Figures 4A, B** **)** and I*κ*Bα (**Figures 4A, C** **)** signaling were significantly decreased by the supplementation of taxifolin. However, no significant difference was observed in the activation of the MAPK (p38, JNK, and ERK) signaling pathway by taxifolin supplementation (data not shown). These results indicated that taxifolin alleviated intestinal inflammation *via* blocking NF-*κ*B signaling, but not the MAPK signaling pathway. The expressions of tight junction (TJ) proteins occludin (**Figures 4A, D** **)** and claudin 1 (**Figures 4A, E** **)** were significantly increased by the taxifolin supplementation, indicating that the gut barrier was enhanced by taxifolin to suppress colitis.

### Gut Microbiota Profiling

**Figure 5:**
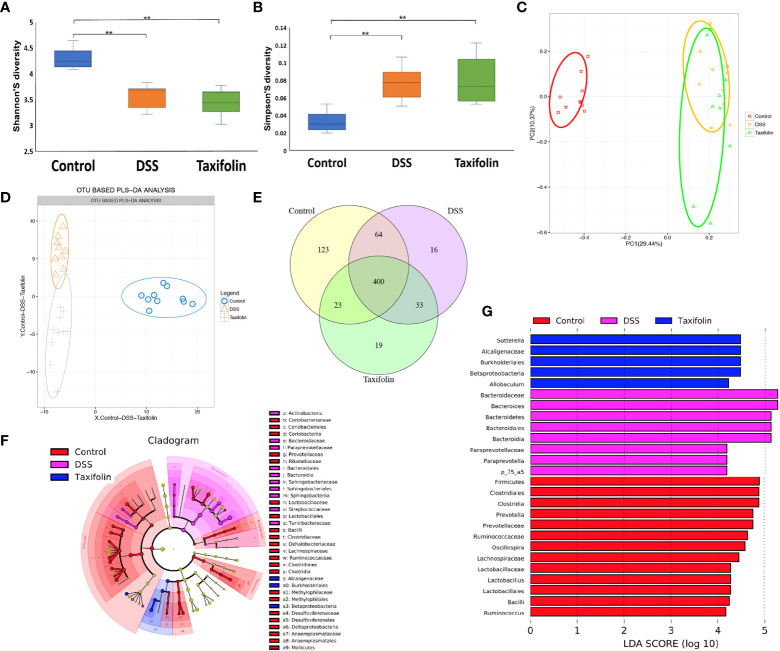
Taxifolin altered the composition of the colon microbiota in colitis mice. **(A**, **B)** Alpha diversity was estimated by the Shannon and Simpson indexes; **(C**, **D)** Principal component analysis (PCA) plot and partial least squares discriminant analysis (PLS-DA) of gut microbiota; **(E)** Venn diagram of OTUs; **(F)** Taxonomic cladogram of LEfSe analysis. Different colors indicate the enrichment of the biomarker taxa in the control (red), DSS (pink), and taxifolin (purple) group. The circle from inside to outside means the rank from kingdom to species, and the circle size represents the taxa abundance in the community. **(G)** Linear discriminant analysis (LDA) score for different taxa abundances. Data were expressed as means ± SEM (*n* = 10).**P < 0.01.

The colon microbial community of the mice with different treatments was evaluated by a 16S rDNA phylogenetic method with the OTUs >97% similarity. Alpha diversity of microbial communities was expressed by Shannon and Simpson indexes. Shannon index was significantly reduced (**Figure 5A**), while Simpson index was significantly increased by both DSS and taxifolin treatments (**Figure 5B**). The structural shifts of gut microbiota were analyzed by principal component analysis (PCA) based on uniFrac distance, showing that the microbiota was separated by DSS and taxifolin treatments (**Figure 5C**). Compared with the control and DSS group, the microbiota was clearly separated by taxifolin supplementation using the partial least squares discriminant analysis (PLS-DA) based on OTU (**Figure 5D**). The Venn diagram result showed that 400 universal OTUs were detected out of 678 total OTUs in all samples. There were 123, 16, and 19 unique OTUs in control, DSS, and taxifolin group, respectively (**Figure 5E**). In addition, the key bacterial alterations in the taxonomic cladogram showed that the most dominant bacteria in the microbial communities were *Alcaligenaceae*, *Burkholderials*, and *Betaproteobacteria* as unique cluster markers in taxifolin group (**Figure 5F**). The LDA score for different levels of taxa abundance showed that the DSS treatment increased pathogen bacteria abundances, such as *Bacteroidaceae* and *Bacteroides*. Moreover, the abundances of *Sutterella*, *Alcaligenaceae*, *Burkholderiales*, *Betaprotecteria*, and *Allobaculum* were significantly increased by the taxifolin treatment (**Figure 5G**).

**Figure 6:**
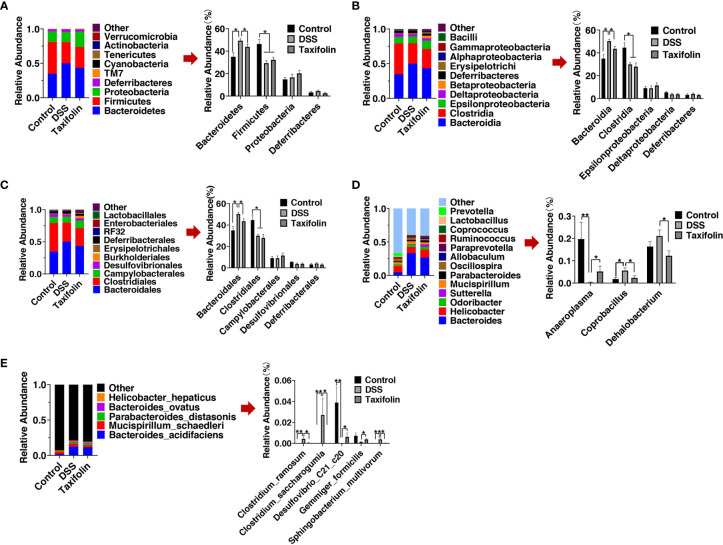
Relative abundance of predominant bacteria was shown at the phylum **(A)**, class **(B)**, order **(C)**, genus **(D)**, and species **(E)** levels. Data were expressed as means ± SEM (*n* = 10). *P < 0.05; **P < 0.01.

The results of microbial composition change of microbial communities showed that *Bacteroidetes* and *Firmicutes* were the main orders at the phylum level, while *Bacteroidia* and *Clostridia* were the main orders at the class level, and *Bacteroidales* and *Clostridiales* were the main orders at the order level (**Figures 6A–C**). At the phylum level, *Bacteroidetes* and the ratio of *Bacteroidetes*/*Firmicutes* were significantly increased by DSS treatment, while these alterations were significantly alleviated by taxifolin supplementation (**Figure 6A**). *Bacteroidetes* and *Firmicutes* were the main phyla, and the relative abundance of *Bacteroidetes* was increased by DSS treatment, while it was significantly restored by taxifolin supplementation (**Figure 6A**). *Becteroidia* at the class level and *Bacteroidetes* at the order level were significantly increased by the DSS challenge, while these shifts were restored by the taxifolin supplementation (**Figures 6B, C** **)**. At the genus level, the abundances of *Coprobacillus* spp. and *Dehalobacterium* spp. were significantly decreased by the taxifolin supplementation to alleviate their alterations caused by DSS. Additionally, the abundance of *Anaeroplasma* spp. was significantly increased by taxifolin (**Figure 6D**). At the species level, the abundances of *Clostridium ramosum*, *Clostridium saccharogumia*, and *Sphingobacterium multivorum* were significantly decreased and the abundances of *Desulfovibrio C21 c20* and *Gemmiger formicilis* were significantly increased by taxifolin supplementation, which restored the changes induced by the DSS challenge (**Figure 6E**).

**Figure 7:**
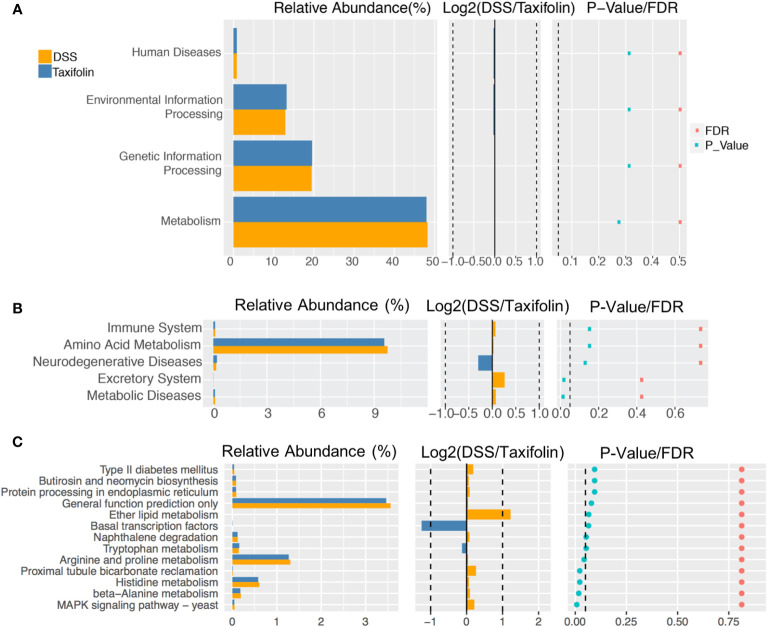
The pathway of different abundances of microflora at KEGG levels 1 **(A)**, 2 **(B)** and 3 **(C)**.

The function of microbial communities was predicted using the PICRUSt software. There was no significant difference of the microbial function files at KEGG level 1 (**Figure 7A**). However, at KEGG level 2, the excretory system and metabolic diseases in the DSS group were decreased significantly by the taxifolin treatment (**Figure 7B**). At KEGG level 3, compared with the DSS group, the functions of microbial communities, including arginine and proline metabolism, proximal tubule bicarbonate reclamation, histidine metabolism, β-alanine metabolism and MAPK signaling, were significantly changed by the taxifolin supplementation (**Figure 7C**).

## Discussion

As a chronic gut inflammation, IBD is a critical worldwide public health problem (1). Many studies have been conducted to investigate the protective effects of plant-derived polyphenols on colitis to limit the side effects of drugs in the treatment of IBD (3). In this study, the flavonoid taxifolin was chosen to explore its effects on mice colitis due to its anti-inflammatory properties (24). However, little is known of the effect of taxifolin on intestinal inflammation and IBD, which is also related to oxidative stress. Various polyphenols with antioxidative activity, such as ellagic acid, procyanidin, AcEGCG and myricitrin, can ameliorate mice colitis *via* anti-oxidant activity and NF-*κ*B signaling (2–5, 7, 8). Therefore, it can be hypothesized that taxifolin with the anti-oxidant activity may function in alleviating intestinal inflammation.

Mice colitis models induced by DSS have been widely used for the investigation of colitis and IBD (1, 27). In these models, DAI value, BW, and colon length are vital markers to evaluate colitis (6, 28). In the present study, higher DAI and shorter colon length were induced by DSS in mice, indicating the mice colitis model was successfully established (29). However, these colitis markers were changed by taxifolin, suggesting that the mice colitis was alleviated by taxifolin. Additionally, a dose of 100 mg/kg BW taxifolin was chosen based on previous reports supporting the safe usage of taxifolin (15, 30).

Cytokines IL-1β, IL-6, TNF-α, and IL-10 play a vital role in modulating chronic intestinal inflammation (1). It has been reported that the increases of TNF-α, IL-1β, and IL-6 can lead to intestinal dysfunction (28). In this study, the decrease of TNF-α and IL-6 productions and the increase of SOD production caused by taxifolin were confirmed by a previous report in liver injury mice (22). Another polyphenol, Ellagic acid, shows similar effects by reducing the expression and production of IL-6 and TNF-α, and MAPK phosphorylation in colitis mice (7, 31). In addition, oxidative stress is another mechanism leading to IBD (32), and the SOD activity can be decreased by the DSS treatment (26, 33). Pre-administration of taxifolin showed protective effects by decreasing pro-inflammatory cytokine production and increasing SOD activity and anti-inflammatory IL-10 production. Similarly, in DSS-induced colitis, anti-inflammatory cytokine IL-10 can modulate intestinal inflammation through a macrophage-ROS axis (1, 32). These results demonstrated that taxifolin could inhibit colitis through mediating intestinal inflammatory cytokines and oxidation resistance.

As the most abundant immunoglobulin in the intestine, SIgA is crucial to mucosal immunity and can protect intestinal membranes with the therapeutic implication for IBD (4). The SIgA concentration was decreased by DSS and increased by the taxifolin treatment, suggesting that the mucosal immunity was enhanced by taxifolin to against DSS damage. This result was consistent with a previous study which shows that the supplement of cranberry proanthocyanins has an increasing effect on the SIgA level to alleviate intestinal inflammation (34). Similarly, the concentrations of immunoglobulins IgA, IgG, and IgM in serum were increased with taxifolin treatment. These alterations of immunoglobulins were consistent with previous studies, such as the supplementations of serine in DSS-induced mice colitis (28) and arginine in piglets (35). These results suggested that dietary taxifolin could alleviate colitis through enhancing immunoglobulin levels.

The mechanisms of antioxidative and anti-inflammatory function of taxifolin include the inhibition of NF-*κ*B (14, 19) and JAK2/STAT3 signaling (36). Taxifolin inhibited inflammation *via* suppressing the activation of NF-*κ*B, which can regulate the gene transcription of inflammatory cytokines (37). Cytokines IL-1β, IL-6, and TNF-α can activate NF-*κ*B phosphorylation in colitis. Therefore, in this study, the decrease of these cytokines caused by taxifolin led to a lower expression of NF-*κ*B phosphorylation. In agreement with a previous study that a flavonoid procyanidin suppressed NF-*κ*B signaling (5), taxifolin also inhibited NF-*κ*B (p65 and I*κ*B*α*) phosphorylation. In DSS-induced mice colitis, the phosphorylation of NF-*κ*B was inhibited by the taxifolin treatment, which can be attributed to its ability to prevent I*κ*Bα degradation (1, 7). The activation of NF-*κ*B and the levels of cytokines in DSS-induced colitis are decreased by oral flavonoid myricitrin (2). Another anti-inflammatory mechanism of taxifolin may be its antioxidative effect on inhibiting COX-2 expression (19). Various studies have shown the protective effect of polyphenols is similar to our study. For instance, green tea-derived polyphenol AcEGCG can protect against DSS-induced colitis by decreasing IL-1β, IL-6, TNF-α, and COX-2 productions and reducing NF-*κ*B (p65 and IκBα) phosphorylation (8). Treatment with polyphenol ellagic acid can inhibit NF-κB expression in colitis (7, 31). Moreover, alga extract with polyphenols suppresses NF-*κ*B phosphorylation in LPS-treated mice (38). Dietary resveratrol treatment ameliorates the increases of secretions of TNF-α and IL-1β and phosphorylation of p65 in DSS-induced colitis mice (13).

The impairment of TJ barrier integrity is another reason for intestinal inflammation. The intestinal physiological barrier is formed by TJ proteins, including claudins, occludin, and ZO-1, which have critical roles in modulating intestinal permeability and IBD pathogenesis (9). In this work, the expression of occludin and claudin-1 in colon was increased by taxifolin supplementation. Similarly, the expression of colonic TJ proteins was also increased by polyphenols naringenin (9), chlorogenic acid (10), and resveratrol (13) to alleviate intestinal inflammation. However, in this study, no significant effect of taxifolin was observed on MAPK phosphorylation in mice colitis (data not shown), indicating that the MAPK signaling is not the mechanism for taxifolin to ameliorate colitis.

Intestinal microbiota plays a critical pathogenic role in chronic inflammation and the alteration of gut oxidative environment can lead to microbiota dysbiosis (39). Intestinal microbial composition and diversity are decreased in colitis mice (40). The colon microbial community composition and diversity were changed by DSS and taxifolin. As the highest relative abundance microbiota, *Bacteroidetes* was increased by DSS and normalized by taxifolin supplementation. In addition, the ratio of *Bacteroidetes*/*Firmicutes* was decreased by the taxifolin treatment. This result is consistent with a previous study, in which the supplementation of polyphenol chlorogenic acid can inhibit *Bacteroides* growth to alleviate intestinal inflammation (41). In another study, the abundances of *Clostridia* and *Firmicutes* in DSS-induced colitis mice are also increased by serine supplementation (28). The increase of *Bacteroidetes* and the ratio of *Bacteroidetes*/*Firmicutes* are the indicators of IBD (27, 28). The decrease of *Bacteroides* abundance could explain the decreased colitis severity with taxifolin supplementation as *Bacteroides* are associated with intestinal inflammation (27, 42). These results suggested that taxifolin helped to alleviate the decrease of microbial diversity shifts induced by DSS challenge.

The supplementation of taxifolin also shifted the microbiota composition and function. The 16S rDNA sequencing suggested that taxifolin increased the richness and shaped the microbiota composition, such as the restoration of *Clostridium ramosum*, *Clostridium saccharogumia*, *Sphingobacterium multivorum*, *Desulfovibrio C21 c20*, and *Gemmiger formicilis* (producing lactate). The DSS treatment can increase the abundance of *Clostridium* species (43), which can induce TNF-α release and colitis (44). Therefore, the decrease of *Clostridium* spp. by taxifolin results alleviate mice colitis, as the effect of pinocembrin (29) and sasa quelpaertensis leaf extract (43). This composition alteration was consistent with a previous report that taxifolin can inhibit the abundances of *S. aureus CNRZ3* and *L. monocytogenes ATCC 19115* (25). In this study, taxifolin inhibited *E. coli* and increased *Allobaculum* abundance, which is beneficial for gut health against inflammation. Similar effects have been observed when treated with soy milk and fiber treatment (45). In addition, taxifolin affected the metabolism of gut microbiota, such as MAPK signaling and amino acid metabolism, suggesting these pathways are the potential targets for taxifolin to manage intestinal inflammation and colitis. There are numbers of studies of dietary polyphenols on colitis (3); however, their effects on gut microbiota are still lacking. Therefore, the modulating effects of taxifolin and other plant polyphenols on gut microbiota need to be further explored.

In conclusion, these results support the hypothesis that oral administration of taxifolin can protect against mice colitis through alleviating mucosa damage and inflammatory responses, inhibiting NF-*κ*B signaling, improving TJ barrier function, and normalizing the gut microbiota. This study provides new insights into the biological functions and therapeutic potential of taxifolin in the treatment of IBD. In the future, the role and the underlying mechanism of the selected gut bacteria in alleviating colitis need to be explored using fecal microbiota transplantation technology or germ-free mice.

## Data Availability Statement

The raw data supporting the conclusions of this article will be made available by the authors, without undue reservation.

## Ethics Statement

The animal study was reviewed and approved by Laboratory Animal Ethical Commission of Huazhong Agricultural University.

## Author Contributions

LM and QX designed the experiment. JH, MH, LZ, YG, and QX performed the animal trials, and sample and data analysis. JH, MH, and QX wrote the manuscript. LM and QX revised the manuscript. All authors contributed to the article and approved the submitted version.

## Funding

This work was supported by grants from the State Key Laboratory of Animal Nutrition (2004DA125184F1906), the Open Project Program of Key Laboratory of Feed Biotechnology, and the Fundamental Research Funds for the Central Universities (2662019QD021).

## Conflict of Interest

The authors declare that the research was conducted in the absence of any commercial or financial relationships that could be construed as a potential conflict of interest.
